# Supplementary material for: Mental health in Ireland during the Covid pandemic: Evidence from two longitudinal surveys
Source: PLoS One. 2024 Mar 7;19(3):e0279526. doi: 10.1371/journal.pone.0279526 (PMC10919714; doi:10.1371/journal.pone.0279526)
Supplement: Attachment — Submitted filename: Review_Comment_PONE-D-22-33706.docx [file pone.0279526.s001.docx]

**Mental Health in Ireland During the Covid Pandemic: Evidence from Two Longitudinal Surveys**

In this article, author made an attempt to examine the change in mental health for two groups: young adults aged around 23 at the time of onset of Covid (the 1998 cohort) and a sample of principal carers (PCs) of children who were aged 13 at the onset of Covid (the 2008 cohort). He/she used data from two longitudinal surveys in Ireland and mental health as measured by the Centre for Epidemiological Studies Depression - 8 scale was obtained for the last pre-Covid wave and for the Covid wave. The Shapley decomposition was applied to measure the change in the fraction depressed. Concentration index and generalized concentration index were used to investigate the gradient of mental health with respect to equalized income and maternal education. Transition matrices were calculated for both study groups and in the case of the 1998 cohort the analysis was stratified by gender. The article is well written and organized. The topic is interesting and innovative but some revisions are needed according to the following comments.

**Comments**

1. In abstract (methods section), author cannot mention what statistical tools has used. It should mention in this section.
2. The author indicated the advantages of this study are that he/she used larger dataset than others studies in Ireland and measured the mental health of same people both before and after Covid. Research gap is not completely clear to me. Please increase the research gap as much as possible.
3. In Table 1, content of the table is vague according to the table title.
4. The authors measured socioeconomic gradient by income and maternal education. Why only choose two variables, income and maternal education? Why not others variables that’s impact on mental health during the Covid pandemic?
5. How can author check the significance of difference between pre-Covid and post-Covid depression rate (Table 3)? What statistical test was performed?
6. How can author compute the transition matrix? Please mention the procedure in the methodology?
7. What are the differences between your study findings and the previous study findings (from literature review)?
8. What are the future implications of this study (since Covid pandemic has already passed)? Please include a recommendation section on the basis of the study findings.
9. Conclusions section is absent in the manuscript. Why?

**Decision**

Major revisions are needed to publish the article in the PLOS ONE journal. I recommend that this paper can be published if author(s) can incorporate the above mentioned comments into the manuscript.
