# Supplementary material for: Mental health in Ireland during the Covid pandemic: Evidence from two longitudinal surveys
Source: PLoS One. 2024 Mar 7;19(3):e0279526. doi: 10.1371/journal.pone.0279526 (PMC10919714; doi:10.1371/journal.pone.0279526)
Supplement: Attachment — Submitted filename: Response to reviewers.docx [file pone.0279526.s002.docx]

Firstly, I would like to thank both reviewers for taking the time to read my paper and for offering very helpful comments. Below I give my response to these comments.

**Reviewer 1**:

*"Following the approach outlined in Kolenikov and Shorrocks
(2005) we take the average of the two effects respectively thus giving a growth effect...."
Why do you take the average of the two impacts, and is there any justification for applying them?*

**Response:** Thank you. The change in depression rates is decomposed into that part which arises from a change in the average level of depression (as measured by the average of the CESD scores) and from a change in the distribution of the CESD scores, which we label the growth and distribution effects respectively. Such a decomposition requires the choice of a “base” configuration for both of these effects. The choices are (i) to calculate the CESD growth effect with the initial CESD distribution held constant, and to calculate the CESD distribution effect holding the mean CESD at the final level or (ii) to calculate the CESD growth effect with the final CESD distribution held constant, and to calculate the CESD distribution effect holding the mean CESD at the initial level. The choice between approach (i) or (ii) is arbitrary but the precise decompositions will vary. The standard solution to this (as followed in Kolenikov and Shorrocks, 2005, for example) is to take the average of the two effects. I have edited the text to try to make this clearer.

**Reviewer 2**

*In abstract (methods section), author cannot mention what statistical tools has used. It should mention in this section*.

**Response:** I have now included some new sentences which describe the statistical analysis which is carried out.

*The author indicated the advantages of this study are that he/she used larger dataset than others studies in Ireland and measured the mental health of same people both before and after Covid. Research gap is not completely clear to me. Please increase the research gap as much as possible.*

**Response:** Thank you. The principal advantage of this study is that we can measure mental for the same people before and during the pandemic. Thus we can identify the change in mental health which occurs during the pandemic. Other studies which monitored mental health during the pandemic, with the exception of Smyth and Murray, provided no information regarding mental health before the pandemic. Thus, using just those studies, there is no way of knowing whether there is a change in mental health following the pandemic, or whether those who suffer from mental health problems in the pandemic had previous conditions. The larger dataset also provides clear advantages in terms of statistical significance and precision of effect sizes. I have inserted extra text to explain this.

*In Table 1, content of the table is vague according to the table title.*

**Response:** Thank you, I have edited the table title and I think it is clearer now.

*The authors measured socioeconomic gradient by income and maternal education. Why only choose two variables, income and maternal education? Why not others variables that’s impact on mental health during the Covid pandemic?*

**Response:** The analysis of how any health condition varies by socioeconomic status (SES) is a standard exercise in health economics and epidemiology. To carry out such analysis we need a measure of SES which is measured accurately and which is clearly ranked (ranking is necessary to calculate concentration indices), in the sense that given any two observations it is always possible to rank one as having a higher SES than the other, or that they are equal. While there may be other variables which influence mental health during the pandemic, if they are not clearly a measure of SES and cannot be ranked (e.g. social class is a measure of SES but it can be difficult to provide a clear ranking). The two variables available in the Growing Up in Ireland survey (GUI) which are best suited for measuring SES are maternal education and after-tax income. I have elaborated further on this in the text.

*How can author check the significance of difference between pre-Covid and post-Covid depression rate (Table 3)? What statistical test was performed?*

**Response:** This analysis was carried out using the DASP programme in Stata (DASP: Distributional Analysis Stata Package, Abdelkrim Araar, Jean-Yves Duclos, Université Laval, PEP and World Bank, December 2021). The statistical test is a standard difference in proportions test, which I now mention in the text.

*How can author compute the transition matrix? Please mention the procedure in the methodology?*

**Response:** The transition matrix is an empirical transition matrix and the graphical version of it is calculated directly from the data using the *tabplot* command in Stata. The underlying data used to construct it can be obtained from a cross tabulation of the data by CESD score and GUI wave. The matrix is a *nxn* matrix, the column shows the fraction of the sample with that CESD score pre-Covid, and the row indicates the fraction of the sample with that CESD score post-Covid. The entries along the main diagonal of the matrix indicate the fraction whose CESD score does not change pre and post Covid. I have edited the methodology section to explain this.

*What are the differences between your study findings and the previous study findings (from literature review)?*

**Response:** Thank you. Our study differs from other Irish studies mentioned in the literature review in explicitly taking account of the dynamics of depression by looking at the change in CESD scores pre and post Covid. It also explicitly examined the socioeconomic gradient associated with CES-D8 scores pre and post pandemic. The study also differs in that it decomposes the overall change in depression into growth and distribution effects These issues are covered in the discussion and I have expanded on this in the text.

*What are the future implications of this study (since Covid pandemic has already passed)? Please include a recommendation section on the basis of the study findings.*

**Response:** Thank you. The study is primarily descriptive and I am wary of drawing strong policy conclusions from it, especially as the circumstances of Covid-19 may be unique and even if we have future pandemics (which seems likely) their precise nature may differ from Covid 19. I have included a new paragraph at the end of the “Discussion” section which covers these issues.

*Conclusions section is absent in the manuscript. Why?*

**Response:** Thank you. The conclusion section was effectively the last paragraph of the “Discussion” section. I have now re-organised this so that there is a separate, short, conclusions section.
