# Supplementary material for: Mental health in Ireland during the Covid pandemic: Evidence from two longitudinal surveys
Source: PLoS One. 2024 Mar 7;19(3):e0279526. doi: 10.1371/journal.pone.0279526 (PMC10919714; doi:10.1371/journal.pone.0279526)
Supplement: Attachment — Submitted filename: Response to reviewer 3.docx [file pone.0279526.s003.docx]

Thank you for taking the time and trouble to read my manuscript. The email from the editor indicated that more information was requested regarding exclusion criteria for observations. I have tried to make this as clear as possible: a balanced panel was used i.e. only observations which were available for the Covid and the last pre-Covid survey were used. In addition it was not possible to use the children from the 2008 cohort as different mental health measures were collected in the Covid survey and in the last pre-Covid survey, so a comparison was not possible. I now state this more explicitly in the text.

Below I give my response to the specific comments raised by referee 3 and where I have not fully complied with the request, I have indicated why not.

I include the comment in italics and my response in non-italic font.

*The sentence beginning with…’For mothers from the 2008 cohort..’ may be recast to read better e.g..**For mothers from the 2008 cohort, a gradient was observed during the pre-COVID-19 pandemic period with poorer mental health status for lower-income and less educated mothers.

The conclusion must be more comprehensive than just two lines. What are the implications of findings from this study?*

**Response:** Thank you, I have incorporated these edits and extended the “conclusion” section.

*Paragraph 3, PCs should be put in full at the first mention in the introduction. Definition appearing in the abstract doesn’t count here.*

**Response:** Thank you, this edit has been carried out.

*The discussion of GUI data in the introduction is too much. Most of the aspects of the GUI data should be moved to the methods section. A mention in one or two lines of the data at the end of the introduction section may be appropriate.*

**Response:** Thank you. I have removed most of the discussion of the data to the Methods section.

*Research design: this study cannot be cross-sectional because the data collection points are more than one over time. Cross-sectional designs entail a snapshot i.e gathering information at a single point in time.*

**Response:** Thank you, I replaced the words “cross-sectional” with “longitudinal”.

*Mentioned that the CES-D scale has been used in several publications previously. It would be good to cite one or two of these.*

**Response:** Thank you, I have included two extra references to cover this.

*The presentation of literature can be improved. The author may not report extant studies one by one. Synthesis of information of information in a more concise and comprehensive manner is recommended.*

**Response:** Thank you, I have re-drafted this section along the lines suggested. The review now covers evidence for the mental health effects of the initial stages of the pandemic, for the later stages of the pandemic, and also some studies for Ireland.

*The author can either be passive or use the first person in the presentation since they are single author. The use of ‘we’ and ‘our’ as in ‘our paper’ doesn’t make sense.*

**Response:** Thank you. I think this could be viewed as mostly a matter of style. The use of the plural pronoun (we) rather than singular (I), even with a single author, while unusual, is not unprecedented. However, having no strong views on the matter, I have changed to passive mode.

*Table titles are to be put on top of the table not under it. Additionally, it must include the total number of respondents (N = ?)*

**Response:** Thank you, this has been done. I have interpreted the comment regarding sample size as applying to table 1. I have not replicated this information for other tables.

*Table 1 is confusing. The first part is relating maternal education and gender. Doesn’t maternal education entail only women?*

**Response:** Thank you. I have now changed this to “Principal Carer’s education” and have done this throughout the manuscript.

*Figures 10 and 11 mentioned are not appearing in the text.*

**Response:** I am sorry, I think this comment may be mistaken. Figures 10 and 11 appear in the file I submitted “Mental Health in Ireland – figures only.

*How does the methods used control for the effects of other variables to the mental health status?*

**Response:** The methods used here are not regression methods and hence controls for a range of covariates are not used. The analysis is stratified by gender and principal carer’s education and the calculation of the concentration indices also shows how the dependent variable varies across income. In addition, as this is longitudinal data, the analysis is automatically controlled for time invariant variables.

*May need to work on the language used to reflect the professionalism required in publications of this nature. Words such as ‘alas’ ‘disturbing’ could be edited out.*

**Response:** Thank you. Again, perhaps a matter of style, but I have made the edits suggested.

*In paragraph 1, the contribution of the study cannot be availability of the data. The data is already available whether or not the study was conducted!*

**Response:** Thank you, you are quite right! I have edited this to saying that it is the *use* of these measures which is the contribution. To the best of my knowledge no other study has exploited this feature of GUI data.

*Discussion reports the findings more than it discusses the implications of the findings. Also no proper engagement with the literature in the field*.

**Response:** I think it is fair to say that most discussions usually start off with a brief recap of the main results, but then adding context and discussion. The decision to be conservative in terms of reference to previous studies in the literature arises because of the belief that Covid 19 was an event with little or no precedent. However, I have made some edits which have put the results of this study in the context of other literature in the area. Thus there is now a greater stress on the unusual finding that there was little if no socioeconomic gradient associated with the deterioration in mental health. I have also discussed my results in the context of other results regarding differences in mental health by gender e.g. the studies by Vloo et al and also Riecher-Rössler.

In terms of the implications of the findings, I think I have discussed these but I have been reluctant to stress them too much as I believe it is still unclear as to how generalizable results from the Covid pandemic will be. One of the lessons of Covid was that the experience from some previous public health epidemics such as influenza were of limited help. One of the main contributions of this paper is to show, in terms of mental health at least, what sort of data should be collected on a regular basis and which could be of use for future pandemics, and I have included an extra sentence stressing this.
